# Effects of *SLCO1B1* and *SLCO1B3* Genetic Polymorphisms on Valsartan Pharmacokinetics in Healthy Korean Volunteers

**DOI:** 10.3390/jpm11090862

**Published:** 2021-08-30

**Authors:** Gonjin Song, Jee-Eun Chung, Jeong Yee, Kyung-Eun Lee, Kyungsoo Park, Hye-Sun Gwak

**Affiliations:** 1 College of Pharmacy and Graduate School of Pharmaceutical Sciences, Ewha Womans University, 52 Ewhayeodae-gil, Sedaemun-gu, Seoul 03760, Korea; songgonjin@naver.com (G.S.); jjjhello1@naver.com (J.Y.); 2 Institute of Pharmaceutical Science and Technology, College of Pharmacy, Hanyang University, 55 Hanyangdaehak-ro, Sangnok-gu, Ansan 15588, Korea; jechung@hanyang.ac.kr; 3 College of Pharmacy, Chungbuk National University, 660-1, Yeonje-ri, Osong-eup, Heungdeok-gu, Cheongju 28160, Korea; kaylee@cbnu.ac.kr; 4 Department of Pharmacology, Yonsei University College of Medicine, 50-1 Yonsei-ro, Seodaeemun-gu, Seoul 03722, Korea

**Keywords:** valsartan, *SLCO1B1*, *SLCO1B3*, Single nucleotide polymorphism, pharmacokinetics

## Abstract

**Purpose:** This study aimed to examine OATP1B1 (*SLCO1B1*) and OATP1B3 (*SLCO1B3*) on the pharmacokinetics of valsartan. Twenty-five subjects were genotyped for 16 single-nucleotide polymorphisms of the *SLCO1B1* and *SLCO1B3* genes. **Methods:** After a single dose of 160 mg of valsartan was orally administered to healthy male volunteers, drug concentrations were assayed up to 48 h. The 25 subjects were genotyped for 16 single-nucleotide polymorphisms (SNPs) of the *SLCO1B1* and *SLCO1B3* genes. Subjects were classified into groups according to their SLCO1B1*1B haplotype; 23 subjects were carriers of SLCO1B1*1B and two subjects were included in the reference group with SLCO1B1*1A/*1A. Alternations of the splicing factor-binding site pattern caused by the given mutation were evaluated with the Human Splicing Finder (HSF) 3.1. **Results:** The subjects who carried SLCO1B1*1B showed a 2.3-fold higher clearance than those without the *1B haplotype. Mean C_max_ and AUC_inf_ were reduced by 45% and 54%, respectively, in the SLCO1B1*1B genotype group compared to the reference group with the *1A/*1A genotype (*p* < 0.01). The carriers of the rs4149153 T allele of *SLCO1B3* had a 27% lower mean C_max_ and a 1.5-fold higher Vd compared to homozygotic CC carriers (*p* < 0.05). In a combined analysis of *SLCO1B1* and *SLCO1B3*, subjects not carrying *SLCO1B1* *1B and carrying *SLCO1B3* rs4149153 T allele showed a 1.6-fold higher clearance than those with the other genotypes, whereas mean C_max_ and AUC_last_ were reduced by 35% and 42%, respectively (*p* < 0.05), in the subjects. HSF 3.1 analysis showed that rs4149153 could cause alterations of the acceptor splice site (TAAATACTAAAGAC to TAAATATTAAAGAC) with scoring change (from 72.57 to 71.92, difference = −0.9). **Conclusion:** It was found that plasma exposure to valsartan is significantly decreased in SLCO1B1*1B carriers and carriers of the rs4149153 T allele of *SLCO1B3*, possibly as a result of increased hepatic uptake.

## 1. Introduction

Valsartan is an orally active angiotensin receptor blocker often prescribed to treat hypertension. It selectively inhibits the binding of angiotensin II to the angiotensin type 1 receptor in many tissues, including vascular smooth muscle and the adrenal gland, and blocks vasoconstriction and aldosterone-secreting effects of angiotensin II. Valsartan undergoes a minor degree of metabolism involving 4-hydroxylation, whereas 85% of orally administered valsartan is excreted in an unchanged form [1]. At physiologic pH, valsartan exists as a di-anion and is selectively distributed to the liver. Because of this negative charge, the hepatic uptake of valsartan through anion transporters is likely to be a major pathway for drug disposition. It has been reported that approximately 70% of the total clearance of valsartan is accounted for by hepatic clearance [2].

Organic anion transporting polypeptide (OATP) family mediates hepatic uptake of endogenous compounds, drugs, xenobiotics, and anionic peptides [3,4]. Among the OATP family transporters, organic anion-transporting polypeptide 1B1 (OATP1B1) and organic anion-transporting polypeptide 1B3 (OATP1B3) are exclusively expressed in human hepatocytes [5]; it suggests that two transporters play a crucial role in the hepatic uptake and clearance of organic compounds [6]. As valsartan has an anionic carboxyl moiety, it is efficiently taken up into hepatocytes by OATP family transporters [7]. Yamashiro et al. reported that valsartan was transported by OATP1B1- and OATP1B3-expressing HEK293 cells [8], and Hanna et al. showed that OATP1B1 contributed 27% of hepatic uptake of valsartan, whereas OATP1B3 contributed 73% [9]. In in vitro kinetic study with transporter-transfected cells, OATP1B1 and OATP1B3 accounted for 55% and 42% of intrinsic hepatic clearance of valsartan, respectively [10]. Taken together, both OATP1B1 and OATP1B3 are major hepatic uptake transporters for valsartan.

OATP1B1, which is encoded by *SLCO1B1*, is a glycoprotein consisting of 691 amino acids [11]. A large number of sequence variants have been found in the *SLCO1B1* gene. Two common SNPs of *SLCO1B1,* A388G (rs2306283) and T521C (rs4149056), which have been associated with altered PK parameters of OATP1B1 substrates, form 4 haplotypes: SLCO1B1*1A (388A–521T), *1B (388G–521T), *5 (388A–521C), and *15 (388G–521C) [12,13,14]. OATP1B3, which is encoded by *SLCO1B3*, shares 80% of the OATP1B1 amino acid sequence and shows overlapping substrate specificities with OATP1B1 [15]. Considering the genetic and functional similarities between OATP1B1 and OATP1B3, both may affect PK [16,17]. However, there is no study on the effects of OATP1B1 and OATP1B3 polymorphisms on valsartan PK. Therefore, in this study, we aimed to evaluate the contribution of the *SLCO1B1* and *SLCO1B3* polymorphisms to valsartan PK parameters in healthy Korean individuals.

## 2. Methods

### 2.1. Study Population

The study population was enrolled from among 50 healthy male volunteers who had participated in a bioequivalence study of a composite product comprised of 160 mg valsartan and 10 mg amlodipine [18]. Among these volunteers, 25 healthy men participated in this study after providing additional written consent for genotyping. Eligible subjects were men between the ages of 20 and 50 years who were within 20% of their ideal body weight, with no congenital abnormalities or chronic disease. Exclusion criteria were: (1) use of prescription drugs or herbal medications within 2 weeks or use of nonprescription drugs within the week before the study that potentially interact with valsartan, and (2) use of drugs that induce or inhibit drug-metabolizing enzymes within the month before the study that potentially interact with the study medications. Vital signs monitoring, physical examination, and routine laboratory tests were performed before the start of the study. The study protocol was approved by the Ethics Committee of the Institutional Review Board (IRB No. 2012-4-0283). Informed consent was obtained from all study subjects before their participation in the study.

### 2.2. Clinical Study

The PK data of the study population were obtained from the previous single-dose study of valsartan [18]_._ Subjects took a 160 mg tablet of valsartan orally with 240 mL of water at 8 a.m. after an overnight fast for 10 h. Venous blood samples were collected into ethylenediaminetetraacetic acid-containing tubes by an indwelling catheter inserted into the forearm at 0 (pre-dose) and 0.5, 1, 1.5, 2, 3, 4, 6, 8, 10, 12, 16, 24, and 48 h after dosing. Blood samples for genotyping were also collected, and genotyping was performed after the end of the study.

### 2.3. Analysis of Valsartan Concentrations and Genotyping

Plasma valsartan concentrations were analyzed with a validated UPLC-MS/MS method as reported in a previous study [18]. Genomic DNA was prepared from blood samples using the QIAamp DNA Blood Mini Kit (QIAGEN GmbH, Hilden, Germany), according to the manufacturer’s standard recommended procedures.

SNPs of *SLCO1B1* and *SLCO1B3* were selected based on other studies and genetic information from the UCSC Genome Browser. Linkage disequilibrium data and minor allele frequency (MAF) data in Japanese and Han Chinese populations from Haploreg ver. 2 and the tagger function within the Haploview v4.2 program were incorporated to assort *SLCO1B1* and *SLCO1B3* gene SNPs. Tag SNPs of the *SLCO1B1* and *SLCO1B3* genes were assigned with a condition of MAF ≥ 30% and an r^2^ threshold of 0.8 in Japanese and Han Chinese populations. In addition, based on the functional effects and previous reports [13,19,20], 11 SNPs (rs12317268, rs4149071, rs4149042, rs2417954, rs4149022, rs4149081, rs2306283, rs4149085, rs4149032, rs4149031, and rs4149056) were selected for *SLCO1B1* and 5 SNPs (rs4149118, rs7311358, rs10841661, rs4149153, and rs11045585) were selected for *SLCO1B3*. Genotyping of *SLCO1B1* and *SLCO1B3* polymorphisms was conducted with a single-base primer extension assay using ABI PRISM SNaPShot Multiplex kits (ABI, Foster City, CA, USA) according to the manufacturer’s recommendations.

### 2.4. Pharmacokinetic Analysis

PK parameters were calculated using actual sampling times. Maximum blood concentration (C_max_) and time to maximum concentration (T_max_) were obtained from the observed data. The area under the plasma concentration-time curve from time zero to the time of the last concentration (AUC_last_) was calculated using the linear trapezoidal rule. The AUC from time zero to infinity (AUC_inf_) was the sum of AUC_last_ and C_last_/k_e_, where C_last_ is the last quantifiable concentration and k_e_ is the terminal elimination rate constant; the half-life was 0.693/k_e_. Plasma concentrations during the terminal phase were fitted to a log linear line by the least squares method to obtain the k_e_. PK parameters were analyzed by a non-compartmental method using WinNonlin5.3 (Pharsight Corporation, Mountain View, CA, USA).

### 2.5. In Silico Analysis

To predict the possible effects of given variants on splicing, different computational tools were used. Netgene2 and Splice Site Prediction by Neural Network (NNSPLICE) were used for splice site predictions [21,22]. Alternations of the splicing factor-binding site pattern caused by the given mutation were evaluated with the Human Splicing Finder (HSF) 3.1 [23]. We used the default threshold values, and a score for a given sequence was considered to be potentially significant if it was above the threshold value.

### 2.6. Statistical Analysis

All PK data were expressed as mean ± SD. The median difference with 95% confidence interval was calculated with the Hodges-Lehmann approach and the method of Moses [24]. Differences in PK parameters among the genotype groups were evaluated using the Kruskal-Wallis test for three-group comparison and the Mann Whitney rank sum test for two-group comparison following normality and equal variance tests; *p* values < 0.05 were considered statistically significant. Statistical analyses were performed using SPSS 20.0 (International Business Machines Corp., New York City, NY, USA).

## 3. Results

All subjects who completed blood sampling as scheduled were enrolled, and only those subjects who gave their consent for the genotyping study were included. Mean age, weight, and height of the 25 subjects were 26.8 ± 5.9 years, 67.9 ± 8.22 kg, and 174.2 ± 4.79 cm, respectively. There were no statistically significant differences in PK parameters according to clinical characteristics (Table 1). The mean PK parameter values were: AUC_inf_ 33.23 ± 14.53 h·μg/mL, CL/F 5.68 ± 2.39 L/h, C_max_ 4.39 ± 1.45 μg/mL, t_1/2_ 6.86 ± 3.02 h, k_e_ 0.11 ± 0.04 h^−1^, T_max_ 3.20 ± 1.25 h, and V_d_/F 51.29 ± 18.40 L. According to the *SLCO1B1* haplotype, the numbers of carriers of SLCO1B1*1A/*1A, *1A/*1B, *1B/*15 and *1B/*1B were 2, 9, 6, and 8, respectively. None of the study subjects had the SLCO1B1*5 haplotype.

As shown in Table 2, subjects who carried SLCO1B1*1B showed more than 2.3-fold higher clearance (CL/F) compared to those with *1A/*1A (*p* < 0.01), resulting in a significantly lower AUC, including AUC_last_ (*p* < 0.05) and AUC_inf_ (*p* < 0.01). V_d_/F increased by 87%, whereas C_max_ decreased by 45% in subjects with the SLCO1B1*1B haplotype, compared to those with *1A/*1A (*p* < 0.01). There were no significant differences in the T_max_, t_1/2_, and k_e_.

In the case of *SLCO1B3*, V_d_/F increased by 50% (*p* < 0.05), whereas C_max_ decreased by 27% (*p* < 0.05) in subjects who were T allele carriers of rs4149153 compared to those with CC genotype. When subjects were classified according to *SLCO1B3* haplotype on the basis of nucleotide base differences in IVS4+76/699/IVS12-5676 (rs4149118/rs7311358/ rs11045585), there was no statistically significant difference between those haplotypes (Table 3).

**Figure 1:**
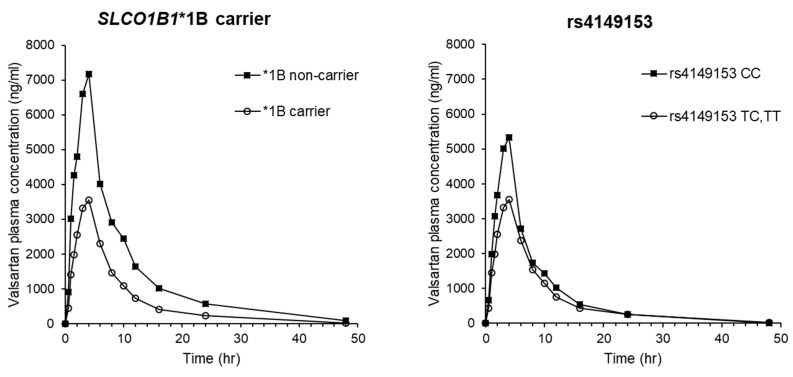
Mean plasma concentration-time profiles of valsartan after a single oral dose of valsartan in 25 healthy Korean male participants with SLCO1B1*1B haplotypes and rs4149153 in *SLCO1B3*.

To predict a splicing effect, analysis by HSF 3.1 showed that SLCO1B3 rs4149153, which had significant associations with C_max_ and Vd/F of valsartan, could cause alterations of the acceptor splice site (TAAATACTAAAGAC to TAAATATTAAAGAC) with scoring change (from 72.57 to 71.92, difference = −0.9). These results are shown in Table 4. The results generated by Netgene2 and NNSPLICE did not show the presence of altered splicing donor or acceptor by rs4149153. By HSF 3.1, rs4149153 was predicted to alter potential branch points, enhancer motifs, and silencer motifs. This mutation decreased the branch points calculation score from 73.16 to 49.32, breaking a potential branch point (threshold: 67). Two types of enhancer motifs (Predicted Exonic Splicing Enhancers octamers and Exon-Identity Elements) were disrupted by rs4149153 (CTAAAGAC and AATAC, respectively). This mutation was also predicted to break one Sironi’s Motif 1 (CTAAAGAC) and three Predicted Exonic Splicing Silencers octamers (TTAAATAT, TAAATATT, and ATATTAAA) [25]. The mean plasma concentration-time profiles of valsartan after administration to subjects with SLCO1B1*1B haplotypes and rs4149153 of *SLCO1B3* are shown in Figure 1. The profiles for rs2306283 and rs4149056, which consist of SLCO1B1*1B haplotype, are shown in Figure S1.

## 4. Discussion

The main findings of this study are that SLCO1B1*1B gene polymorphism and rs4149153 of the *SLCO1B3* gene can influence valsartan PK. Subjects who carried SLCO1B1*1B showed higher CL/F and lower AUC values (AUC_last_ and AUC_inf_) compared to those without the *1B haplotype. The carriers of the rs4149153 T allele in *SLCO1B3* had a 27% lower mean C_max_ and 1.5-fold higher Vd/F compared to homozygotic CC carriers.

A388G (rs2306283, Asn130Asp) and T521C (rs4149056, Val174Ala) are the most frequently studied SNPs related to drug disposition. These polymorphisms have been mainly studied with respect to statins, which are the substrates of OATP1B1. The functional effect of the SLCO1B1*1B haplotype, which is an A388G allele, has been variously reported in different in vitro studies showing increased activity, decreased activity, or no change in the activity [26]. This may be because of substrate-specific effects or differences in the experimental conditions. The inconsistent results of the *1B haplotype also have been reported in clinical studies. SLCO1B1*1B alleles showed accelerated transporter activity and were associated with decreased plasma concentrations of pravastatin. A study showed that the AUC of pravastatin was 35% lower in participants with the *1B/*1B genotype than in participants with the *1A/*1A genotype [19]. In contrast, the SLCO1B1*1B haplotype was associated with increased plasma concentrations of pitavastatin and unchanged AUC of rosuvastatin in Asian populations [27,28,29].

It has been reported that the 521C allele is associated with decreased OATP1B1 activity, and the *5 and *15 haplotypes that contain this allele are low-activity haplotypes [30]. The 521C allele has been reported to be associated with decreased hepatic uptake and increased plasma concentrations of statins including atorvastatin, pitavastatin, pravastatin, rosuvastatin, and simvastatin [14,27,31,32,33]. Through various in vitro and in vivo studies of the *SLCO1B1* haplotype, the effects of the *SLCO1B1* *5 and *15 haplotypes are well documented. Since SLCO1B1*5 is very rarely found in the East Asian population [34], clinical studies in Asian populations have focused on evaluating the increased exposure to substrate and increased risk of toxicity in SLCO1B1*15 carriers [20,34,35]. However, our study did not show significant differences in any valsartan PK parameters between subjects with and without *15.

It has also been reported that the relative contributions of OATP1B1 and OATP1B3 to the hepatic uptake of valsartan are similar [7]. In our study, subjects who carried the T allele of rs4149153 showed lower AUC values (AUC_last_ and AUC_inf_) and higher CL/F and V_d_/F as compared to subjects with the CC genotype (*p* < 0.05). The SNP of rs4149153 is located in an intron region, which is not thought to be involved in protein production. However, intronic regions have the potential to affect mRNA splicing and alter protein expression or action [36,37]. In our *in silico* analysis, although no significant splicing motif alteration was detected, a few site broken variations and new sites were detected. Therefore, rs4149513 may be a candidate SNP affecting valsartan PKs.

To the best of our knowledge, this is the first evaluation of the association between *SLCO1B1* and *SLCO1B3* gene polymorphisms and valsartan PKs. However, this study had some limitations. The sample size was too small to yield statistically significant results. Our study population consisting of only male subjects precluded analysis of gender differences. Since this study is not a hypothesis-generating study but is intended to find candidate genes affecting valsartan PKs, multiple test correction was not performed. Therefore, it should be implemented with a caution with the risk of false-positive results and needs to be verified by further replication studies.

In conclusion, we investigated that plasma exposure to valsartan is significantly decreased in SLCO1B1*1B carriers and carriers of the rs4149153 T allele of *SLCO1B3*, possibly as a result of increased hepatic uptake. As these associations have not been proven in cell lines and preclinical models, further in vitro and in vivo studies are needed to strengthen the findings of our clinical researches.

## Figures and Tables

**Table 1:** Effects of baseline characteristics on valsartan pharmacokinetics.

	N	AUC_last_ (h·μg/mL)	AUC_inf_ (h·μg/mL)	CL/F (L/h)	C_max_ (μg/mL)	t_1/2_ (h)	k_e_ (h^−1^)	T_max_ (h)	V_d_/F (L)
Age (years)									
<25	9	33.54 ± 18.18	36.11 ± 18.96	5.32 ± 2.2	4.79 ± 1.39	7.12 ± 1.82	0.1 ± 0.03	3.11 ± 1.52	50.15 ± 11.62
≥25	13	30.44 ± 11.73	32.05 ± 12.12	5.81 ± 2.55	4.18 ± 1.47	6.96 ± 3.86	0.12 ± 0.04	3.23 ± 1.24	52.85 ± 22.87
D (95% CI)		0.79 (−11.31, 11.46)	1.42 (−8.93, 13.6)	−0.21 (−2.56, 1.6)	0.83 (−0.58, 1.73)	1.03 (−0.91, 2.7)	−0.02 (−0.05, 0.02)	0 (−1.5, 1)	1.41 (−20.2, 15.89)
Body mass index (kg/m^2^)									
<23	13	33.69 ± 16.9	35.73 ± 17.65	5.53 ± 2.75	4.77 ± 1.69	6.46 ± 2.41	0.12 ± 0.03	2.88 ± 1.29	46.55 ± 17.35
≥23	9	28.84 ± 9.95	30.78 ± 10.35	5.73 ± 1.86	3.94 ± 0.85	7.84 ± 3.96	0.1 ± 0.04	3.61 ± 1.32	59.26 ± 19.12
D (95% CI)		3.09 (−8.16, 14.23)	2.74 (−8.41, 16.32)	−0.7 (−2.44, 1.71)	0.79 (−0.5, 2.13)	−1.09 (−2.76, 1.03)	0.02 (−0.02, 0.05)	−1 (−2, 0)	−13.52 (−26.9, 3.06)
Smoking									
Yes	6	29.16 ± 9.21	31.68 ± 10.06	5.59 ± 2.12	4.15 ± 1.35	6.33 ± 1.66	0.12 ± 0.03	2.75 ± 1.08	48.55 ± 13.15
No	16	32.66 ± 16.06	34.47 ± 16.7	5.62 ± 2.53	4.53 ± 1.5	7.29 ± 3.54	0.11 ± 0.04	3.34 ± 1.4	52.95 ± 20.73
D (95% CI)		−0.63 (−12.19, 9.09)	−0.4 (−14.82, 11.41)	0.07 (−2.38, 2.23)	−0.49 (−1.63, 1.02)	−0.4 (−2.66, 2.09)	0.01 (−0.04, 0.04)	0 (−2, 1)	−1.38 (−22.29, 15.03)
Alcohol									
Yes	15	32.33 ± 16.14	34.53 ± 16.78	5.67 ± 2.7	4.23 ± 1.46	7.5 ± 3.65	0.11 ± 0.04	3.43 ± 1.4	53.93 ± 20.4
No	7	30.37 ± 10.66	31.95 ± 11.18	5.49 ± 1.63	4.85 ± 1.41	6.01 ± 1.17	0.12 ± 0.02	2.64 ± 1.03	47.08 ± 14.96
D (95% CI)		1.09 (−10.49, 12.19)	1.03 (−9.05, 14.9)	−0.23 (−2.28, 2.26)	−0.67 (−1.8, 0.81)	0.62 (−0.92, 3.04)	−0.01 (−0.04, 0.02)	0.5 (0, 2)	5.01 (−13.61, 20.49)
Caffeine									
Yes	10	29.46 ± 9.74	31.64 ± 10.67	5.61 ± 1.88	4.28 ± 0.88	6.38 ± 1.63	0.12 ± 0.03	3.6 ± 1.51	48.41 ± 10.35
No	12	33.58 ± 17.58	35.44 ± 18.14	5.62 ± 2.81	4.55 ± 1.81	7.57 ± 3.97	0.11 ± 0.04	2.83 ± 1.09	54.52 ± 23.8
D (95% CI)		−2.14 (−13.82, 9.25)	−1.42 (−14.82, 9.53)	0.21 (−1.86, 2.28)	0.11 (−1.54, 1.13)	−0.09 (−2.64, 1.59)	0 (−0.03, 0.04)	0.5 (0, 2)	−2.42 (−21.37, 11.88)

AUC_last_: area under the plasma concentration-time curve from time zero to the time of the last concentration, AUC_inf_: area under the plasma concentration-time curve from time zero to the infinity, CL: clearance, F: bioavailability, C_max_: maximum plasma concentration, t_1/2_: terminal half-life, k_e_: elimination rate constant, T_max_: time to maximum concentration, V_d_: volume of distribution, D: difference.

**Table 2:** Effects of *SLCO1B1* polymorphisms on valsartan pharmacokinetics.

	N	AUC_last_ (h·μg/mL)	AUC_inf_ (h·μg/mL)	CL/F (L/h)	C_max_ (μg/mL)	t_1/2_ (h)	k_e_ (h^−1^)	T_max_ (h)	V_d_/F (L)
rs12317268									
AA, AG	23	31.48 ± 14.47	33.52 ± 15.07	5.70 ± 2.48	4.37 ± 1.49	7.05 ± 3.08	0.11 ± 0.04	3.22 ± 1.27	52.52 ± 18.68
GG	2	28.74 ± 6.33	29.92 ± 6.74	5.49 ± 1.23	4.73 ± 1.28	4.76 ± 0.63	0.15 ± 0.02	3.00 ± 1.41	37.12 ± 3.49
D (95% CI)		1.18 (−16.20, 24.40)	1.17 (−16.73, 25.15)	−0.28 (−2.67, 4.75)	−0.48 (−2.70, 2.19)	1.46 (−0.59, 6.48)	−0.03 (−0.09, 0.02)	0 (−2, 2)	14.06 (−9.50, 48.06)
rs4149071									
TT	11	35.46 ± 17.26	37.45 ± 17.93	5.16 ± 2.35	4.93 ± 1.74	7.03 ± 3.99	0.12 ± 0.04	3.00 ± 0.89	46.84 ± 22.00
TC, CC	14	27.96 ± 10.12	29.92 ± 10.74	6.09 ± 2.43	3.98 ± 1.08	6.73 ± 2.13	0.11 ± 0.03	3.36 ± 1.49	54.79 ± 14.92
D (95% CI)		5.71 (−4.34, 14.89)	5.25 (−4.84, 17.03)	−0.02 (−0.12, 0)	1.06 (−0.35, 2.18)	−0.4 (−2.57, 1.31)	0.01 (−0.02, 0.04)	0 (−2, 1)	−11.79 (−23.24, 3.98)
rs4149042									
TT, CT	21	32.44 ± 14.74	34.58 ± 15.31	5.53 ± 2.50	4.39 ± 1.52	7.21 ± 3.18	0.11 ± 0.04	3.19 ± 1.32	52.11 ± 19.23
CC	4	25.08 ± 6.65	26.17 ± 6.87	6.46 ± 1.80	4.39 ± 1.24	5.03 ± 0.48	0.14 ± 0.01	3.25 ± 0.96	46.97 ± 14.64
D (95% CI)		5.38 (−4.80, 17.32)	6.71 (−4.66, 18.16)	−1.35 (−3.18, 1.19)	−0.18 (−1.56, 1.60)	1.6 (−0.29, 3.60)	−0.03 (−0.07, 0.01)	0 (−1.5, 1)	2.98 (−12.85, 23.24)
rs2417954									
AA	10	30.85 ± 11.71	32.21 ± 11.90	5.72 ± 2.56	4.53 ± 1.34	6.59 ± 2.21	0.11 ± 0.03	2.75 ± 0.98	51.15 ± 18.33
AG, GG	15	31.53 ± 15.63	33.91 ± 16.41	5.65 ± 2.36	4.31 ± 1.57	7.04 ± 3.52	0.11 ± 0.04	3.50 ± 1.35	51.38 ± 19.09
D (95% CI)		1.31 (−10.06, 9.79)	−0.06 (−11.16, 9.40)	−0.02 (−2.21, 2.08)	0.49 (−0.86, 1.45)	0.06 (−2.14, 1.45)	0 (−0.03, 0.03)	−1 (−2, 0)	−0.74 (−14.57, 15.76)
rs4149022									
AA, GA	22	31.74 ± 14.75	33.82 ± 15.35	5.69 ± 2.54	4.33 ± 1.52	7.12 ± 3.1	0.11 ± 0.04	3.23 ± 1.30	52.83 ± 19.06
GG	3	27.77 ± 4.78	28.91 ± 5.07	5.64 ± 0.91	4.88 ± 0.94	4.95 ± 0.56	0.14 ± 0.02	3.00 ± 1.00	40.02 ± 5.61
D (95% CI)		1.73 (−9.53, 15.44)	4.6 (−9.98, 16.42)	−0.62 (−2.45, 2.96)	−0.87 (−2.20, 1.12)	1.46 (−0.30, 3.84)	−0.03 (−0.07, 0.01)	0 (−1, 2)	12.64 (−7.76, 28.25)
rs4149081									
GG, AG	23	31.48 ± 14.47	33.52 ± 15.07	5.70 ± 2.48	4.37 ± 1.49	7.05 ± 3.08	0.11 ± 0.04	3.22 ± 1.27	52.52 ± 18.68
AA	2	28.74 ± 6.33	29.92 ± 6.74	5.49 ± 1.23	4.73 ± 1.28	4.76 ± 0.63	0.15 ± 0.02	3.00 ± 1.41	37.12 ± 3.49
D (95% CI)		1.18 (−16.20, 24.40)	1.17 (−16.73, 25.15)	−0.28 (−2.67, 4.75)	−0.48 (−2.70, 2.19)	1.46 (−0.59, 6.48)	−0.03 (−0.09, 0.02)	0 (−2, 2)	14.06 (−9.50, 48.06)
rs2306283									
AA, AG	11	34.41 ± 18.13	37.09 ± 18.88	5.51 ± 3.03	4.45 ± 1.89	7.35 ± 4.05	0.11 ± 0.04	3.45 ± 1.51	50.67 ± 24.11
GG	14	28.79 ± 9.51	30.20 ± 9.63	5.82 ± 1.85	4.35 ± 1.08	6.48 ± 1.96	0.11 ± 0.03	3.00 ± 1.02	51.78 ± 13.32
D (95% CI)		4 (−6.71, 13.77)	6.1 (−6.88, 16.49)	−0.72 (−2.44, 1.09)	−0.18 (−1.36, 1.30)	−0.16 (−1.32, 2.59)	0 (−0.04, 0.03)	0 (−1, 2)	−5.69 (−20.19, 11.88)
rs4149085									
TT	13	32.10± 17.38	34.65 ± 18.05	5.80 ± 2.87	4.44 ± 1.77	6.77 ± 1.88	0.11 ± 0.03	3.38 ± 1.54	51.71 ± 17.67
TC	12	30.35 ± 9.61	31.70 ± 9.99	5.56 ± 1.86	4.35 ± 1.09	6.96 ± 4.00	0.12 ± 0.04	3.00 ± 0.85	50.84 ± 19.94
D (95% CI)		−0.33 (−9.53, 9.46)	1.06 (−8.94, 11.25)	−0.18 (−1.94, 2.16)	−0.09 (−1.25, 1.12)	0.65 (−0.99, 2.65)	−0.02 (−0.04, 0.02)	0 (−1, 2)	2.19 (−11.88, 18.78)
rs4149032									
CC	3	46.51 ± 33.02	48.41 ± 34.00	5.72 ± 5.50	5.67 ± 3.11	7.18 ± 2.97	0.11 ± 0.04	2.67 ± 1.15	48.20 ± 34.19
CT, TT	22	29.18 ± 8.96	31.16 ± 9.68	5.68 ± 1.91	4.22 ± 1.11	6.82 ± 3.09	0.12 ± 0.04	3.27 ± 1.27	51.71 ± 16.55
D (95% CI)		21.4 (−15.95, 52.31)	20.15 (−18.29, 51.48)	−1.67 (−4.39, 6.97)	2.52 (−2.06, 4.07)	0.59 (−2.72, 5.03)	−0.01 (−0.06, 0.04)	0 (−2, 1)	−12.03 (−33.67, 40.27)
rs4149031									
CC	14	32.03 ± 16.53	34.22 ± 17.13	5.70 ± 2.62	4.41 ± 1.67	6.90 ± 2.34	0.11 ± 0.04	3.29 ± 1.53	51.63 ± 17.01
CG	11	30.29 ± 10.43	31.97 ± 11.04	5.66 ± 2.18	4.37 ± 1.20	6.81 ± 3.84	0.12 ± 0.04	3.09 ± 0.83	50.86 ± 20.88
D (95% CI)		−0.63 (−10.30, 9.09)	0.36 (−10.56, 10.12)	−0.03 (−2.09, 1.89)	−0.28 (−1.31, 1.22)	0.47 (−0.90, 2.70)	−0.01 (−0.04, 0.02)	0 (−1, 1)	2.84 (−12.69, 18.82)
rs4149056									
TT	18	31.00 ± 10.62	33.13 ± 11.28	5.54 ± 2.39	4.33 ± 1.36	7.12 ± 3.28	0.11 ± 0.04	3.19 ± 1.34	52.42 ± 19.94
CT	7	31.92 ± 21.36	33.50 ± 21.99	6.03 ± 2.55	4.56 ± 1.78	6.19 ± 2.27	0.12 ± 0.03	3.21 ± 1.07	48.40 ± 14.62
D (95% CI)		4.5 (−7.80, 13.64)	5.66 (−8.93, 15.63)	−0.94 (−2.56, 1.72)	−0.18 (−1.58, 1.22)	0.59 (−0.98, 2.71)	−0.01 (−0.04, 0.02)	0 (−1, 1)	1.78 (−12.85, 19.81)
*1A carrier									
Yes	11	34.41 ± 18.13	37.09 ± 18.88	5.51 ± 3.03	4.45 ± 1.89	7.35 ± 4.05	0.11 ± 0.04	3.45 ± 1.51	50.67 ± 24.11
No	14	28.79 ± 9.51	30.20 ± 9.63	5.82 ± 1.85	4.35 ± 1.08	6.48 ± 1.96	0.11 ± 0.03	3.00 ± 1.02	51.78 ± 13.32
D (95% CI)		4 (−6.71, 13.77)	6.1 (−6.88, 16.49)	−0.72 (−2.44, 1.09)	−0.18 (−1.36, 1.30)	−0.16 (−1.32, 2.59)	0 (−0.04, 0.03)	0 (−1, 2)	−5.69 (−20.19, 11.88)
*1B carrier									
Yes	23	28.45 ± 9.42 ^a^	30.38 ± 10.16 ^b^	5.95 ± 2.29 ^b^	4.13 ± 1.17 ^b^	6.74 ± 3.04	0.12 ± 0.04	3.22 ± 1.27	53.27 ± 17.82 ^a^
No	2	63.53 ± 21.03	65.97 ± 21.48	2.56 ± 0.83	7.47 ± 0.33	8.25 ± 3.30	0.09 ± 0.04	3.00 ± 1.41	28.48 ± 2.26
D (95% CI)		−34.41 (−61.39, −10.49)	−35.78 (−63.21, −9.05)	2.93 (0.68, 7.40)	−3.38 (−5.14, −1.60)	−1.62 (−6.14, 2.72)	0.02 (−0.03, 0.09)	0 (−2, 2)	21.83 (2.69, 57.56)
*15 carrier									
Yes	7	31.92 ± 21.36	33.50 ± 21.99	6.03 ± 2.55	4.56 ± 1.78	6.19 ± 2.27	0.12 ± 0.03	3.21 ± 1.07	48.40 ± 14.62
No	18	31.00 ± 10.62	33.13 ± 11.28	5.54 ± 2.39	4.33 ± 1.36	7.12 ± 3.28	0.11 ± 0.04	3.19 ± 1.34	52.42 ± 19.94
D (95% CI)		−4.5 (−13.64, 7.80)	−5.66 (−15.63, 8.93)	0.94 (−1.72, 2.56)	0.18 (−1.22, 1.58)	−0.59 (−2.71, 0.98)	0.01 (−0.02, 0.04)	0 (−1, 1)	−1.78 (−19.81, 12.85)

AUC_last_: area under the plasma concentration-time curve from time zero to the time of the last concentration, AUC_inf_: area under the plasma concentration-time curve from time zero to the infinity, CL: clearance, F: bioavailability, C_max_: maximum plasma concentration, t_1/2_: terminal half-life, k_e_: elimination rate constant, T_max_: time to maximum concentration, V_d_: volume of distribution, D: median difference, CI: confidence interval. *1B carrier: SLCO1B1*1B (c.388G-c.521T). ^a^ *p* < 0.05, ^b^ *p* < 0.01 in Mann-Whitney U test between 2 genotype groups.

**Table 3:** Effects of *SLCO1B3* polymorphisms on valsartan pharmacokinetics.

	N	AUC_last_ (h·μg/mL)	AUC_inf_ (h·μg/mL)	CL/F (L/h)	C_max_ (μg/mL)	t_1/2_ (h)	k_e_ (h^−1^)	T_max_ (h)	V_d_/F (L)
rs4149118									
GG	5	35.58 ± 13.15	36.81 ± 13.40	4.87 ± 1.86	5.38 ± 1.18	6.11 ± 1.19	0.12 ± 0.02	3.20 ± 0.84	41.71 ± 14.06
AG, AA	20	30.18 ± 14.23	32.34 ± 14.98	5.88 ± 2.51	4.15 ± 1.44	7.05 ± 3.32	0.11 ± 0.04	3.20 ± 1.35	53.69 ± 18.86
D (95% CI)		5.71 (−4.34, 14.89)	5.25 (−4.84, 17.03)	−0.02 (−0.12, 0)	1.06 (−0.35, 2.18)	−0.4 (−2.57, 1.31)	0.01 (−0.02, 0.04)	0 (−2, 1)	−11.79 (−23.24, 3.98)
rs7311358									
GG, AG	16	32.94 ± 16.26	34.66 ± 16.76	5.57 ± 2.36	4.57 ± 1.67	6.72 ± 3.45	0.12 ± 0.04	3.41 ± 1.08	48.69 ± 19.81
AA	9	28.27 ± 8.42	30.69 ± 9.73	5.88 ± 2.57	4.07 ± 0.98	7.11 ± 2.22	0.11 ± 0.03	2.83 ± 1.50	55.92 ± 15.57
D (95% CI)		2.18 (−7.57, 13.37)	1.31 (−8.16, 13.6)	−0.2 (−2.21, 2.09)	0.31 (−0.86, 1.77)	−0.85 (−2.71, 0.86)	0.02 (−0.02, 0.05)	1 (0, 2)	−7.99 (−22.39, 7.60)
rs10841661									
CC	14	28.25 ± 10.82	29.87 ± 11.26	6.20 ± 2.58	4.12 ± 1.28	5.92 ± 1.44	0.12 ± 0.03	2.86 ± 0.86	50.24 ± 16.64
CT, TT	11	35.09 ± 16.87	37.51 ± 17.48	5.02 ± 2.05	4.74 ± 1.64	8.07 ± 4.04	0.10 ± 0.04	3.64 ± 1.55	52.62 ± 21.19
D (95% CI)		−4.7 (−14.23, 4.39)	−5.61 (−16.73, 3.93)	0.77 (−0.70, 2.79)	−0.5 (−1.77, 0.65)	−1.05 (−3.69, 0.43)	0.02 (−0.01, 0.05)	−1 (−2, 0)	−1.06 (−17.80, 13.61)
rs4149153									
CC	4	39.32 ± 11.71	40.67 ± 11.84	4.22 ± 1.34	5.69 ± 1.09 ^a^	6.16 ± 1.37	0.12 ± 0.02	3.00 ± 0.82	36.20 ± 7.87 ^a^
TC, TT	21	29.73 ± 14.03	31.82 ± 14.80	5.96 ± 2.47	4.15 ± 1.40	7.00 ± 3.25	0.11 ± 0.04	3.24 ± 1.33	54.17 ± 18.52
D (95% CI)		10.95 (−2.68, 24.99)	9.84 (−5.97, 25.61)	−1.37 (−3.95, 0.70)	1.51 (0.36, 3.08)	0.09 (−2.71, 1.32)	0 (−0.03, 0.04)	0 (−1, 1)	−16.23 (−32.07, −2.15)
rs11045585									
AA	20	30.61 ± 14.84	32.65 ± 15.51	5.89 ± 2.56	4.28 ± 1.59	7.01 ± 3.31	0.11 ± 0.04	3.30 ± 1.33	53.65 ± 19.75
AG	5	33.85 ± 10.40	35.55 ± 10.75	4.84 ± 1.44	4.83 ± 0.63	6.28 ± 1.42	0.11 ± 0.02	2.80 ± 0.84	41.87 ± 6.22
D (95% CI)		−4.46 (−15.57, 8.17)	−4.74 (−16.74, 8.63)	0.66 (−1.24, 3.18)	−0.85 (−1.81, 0.78)	−0.11 (−1.57, 2.71)	0 (−0.04, 0.03)	0 (−1, 2)	12.45 (−7.29, 25.39)
A/A/A carrier									
Yes	20	30.18 ± 14.23	32.34 ± 14.98	5.88 ± 2.51	4.15 ± 1.44	7.05 ± 3.32	0.11 ± 0.04	3.20 ± 1.35	53.69 ± 18.86
No	5	35.58 ± 13.15	36.81 ± 13.40	4.87 ± 1.86	5.38 ± 1.18	6.11 ± 1.19	0.12 ± 0.02	3.20 ± 0.84	41.71 ± 14.06
D (95% CI)		−8.08 (−20.76, 7.80)	−7.78 (−19.20, 8.39)	0.74 (−1.21, 3.30)	−1.25 (−2.64, 0.02)	−0.13 (−1.29, 2.65)	0 (−0.04, 0.03)	0 (−1, 1)	12.6 (−4.79, 28.09)
G/A/G carrier									
Yes	5	33.85 ± 10.40	35.55 ± 10.75	4.84 ± 1.44	4.83 ± 0.63	6.28 ± 1.42	0.11 ± 0.02	2.80 ± 0.84	41.87 ± 6.22
No	20	30.61 ± 14.84	32.65 ± 15.51	5.89 ± 2.56	4.28 ± 1.59	7.01 ± 3.31	0.11 ± 0.04	3.30 ± 1.33	53.65 ± 19.75
D (95% CI)		4.46 (−8.17, 15.57)	4.74 (−8.63, 16.74)	−0.66 (−3.18, 1.24)	0.85 (−0.78, 1.81)	0.11 (−2.71, 1.57)	0 (−0.03, 0.04)	0 (−2, 1)	−12.45 (−25.39, 7.29)
G/A/A carrier									
Yes	1	20.61 ± 0.00	21.39 ± 0.00	7.48 ± 0.00	4.13 ± 0.00	5.91 ± 0.00	0.12 ± 0.00	4.00 ± 0.00	63.75 ± 0.00
No	24	31.70 ± 14.05	33.72 ± 14.62	5.61 ± 2.41	4.41 ± 1.49	6.90 ± 3.08	0.11 ± 0.04	3.17 ± 1.27	50.77 ± 18.61
D (95% CI)		−8.63 (NA)	−11.18 (NA)	2.56 (NA)	−0.03 (NA)	0.18 (NA)	0 (NA)	1 (NA)	17.14 (NA)

AUC_last_: area under the plasma concentration-time curve from time zero to the time of the last concentration, AUC_inf_: area under the plasma concentration-time curve from time zero to the infinity, CL: clearance, F: bioavailability, C_max_: maximum plasma concentration, t_1/2_: terminal half-life, k_e_: elimination rate constant, T_max_: time to maximum concentration, V_d_: volume of distribution, D: median difference, CI: confidence interval, NA: not available. A/A/A carrier: haplotype A/A/A (IVS4+76/699/IVS12-5676; rs4149118/rs7311358/rs11045585) carrier. ^a^ *p* < 0.05 in Mann-Whitney U test between 2 genotype groups.

**Table 4:** Potential splicing regulatory sequences in wild-type and mutant type of rs4149153 by Human Splicing Finder (HSF) 3.1.

Type of Signal	Matrice	Reference Motif	Mutant Motif	Consensus or Motif Value for Reference Sequence (0–100)	Consensus or Motif Value for Mutant Sequence (0–100)	Variation (%)
Splice acceptor site	HSF Matrices	TAAATACTAAAGAC	TAAATATTAAAGAC	72.57	71.92	−0.9
Branch point	HSF Matrices	TACTAAA	-	73.16	49.32	Site broken
Enhancer motif	PESE Octamers	CTAAAGAC	-	27.80	-	Site broken
	EIEs Hexamers	AAATAC	-	-	-	Site broken
		ATACTA	ATATTA	-	-	-
		ACTAAA	ATTAAA	-	-	-
		CTAAAG	TTAAAG	-	-	-
	HSF Matrices (Tra2-β)	AATAC	AATAT	85.42	85.42	0
Silencer motif	Sironi motifs (Motif 1)	CTAAAGAC	-	63.85	-	Site broken
	PESS Octamers	-	TTAAATAT	-	43.54	New site
		-	TAAATATT	-	39.68	New site
		-	ATATTAAA	-	33.51	New site

## Data Availability

The data presented in this study are available on request from the corresponding author.

## Supplementary Materials

The following are available online at https://www.mdpi.com/article/10.3390/jpm11090862/s1, Figure S1: Mean plasma concentration-time profiles of valsartan after a single oral dose of valsartan in 25 healthy Korean male participants with rs2306283 and rs4149056 in *SLCO1B1*.
