# Supplementary material for: Effects of SLCO1B1 and SLCO1B3 Genetic Polymorphisms on Valsartan Pharmacokinetics in Healthy Korean Volunteers
Source: J Pers Med. 2021 Aug 30;11(9):862. doi: 10.3390/jpm11090862 (PMC8467379; doi:10.3390/jpm11090862)

Supplementary Figure S1. Mean plasma concentration-time profiles of valsartan after a single oral dose of valsartan in 25 healthy Korean male participants with rs2306283 and rs4149056 in *SLCO1B1*.

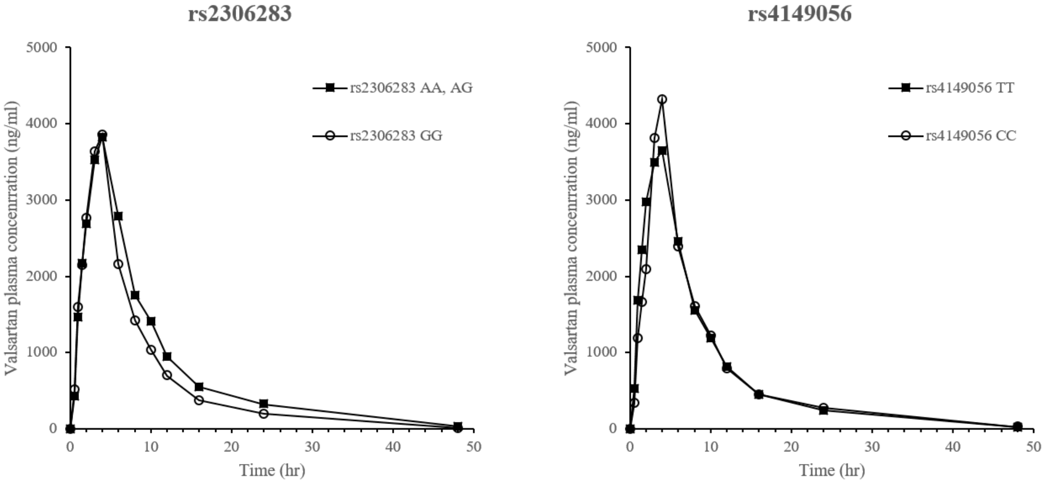

Supplement: Supplementary file 1 [file jpm-11-00862-s001.zip › jpm-1349926-supplementary.pdf]
